# Association of* SDF-1* Gene Polymorphism with Increased Risk of Acute Myeloid Leukemia Patients

**DOI:** 10.31557/APJCP.2021.22.4.1035

**Published:** 2021-04

**Authors:** Doaa Abd Allah M Aladle, Mayada A Ghannam, Shaimaa El-Ashwah, F E I Ghobrial, Metwaly Ibrahim Mortada

**Affiliations:** 1 *Hematology Unit, Department of Clinical Pathology, Mansoura Faculty of Medicine, Mansoura University, Egypt. *; 2 *Clinical Hematology Unit, Department of Internal Medicine, Oncology Center, Mansoura University, Mansoura, Egypt. *; 3 *Medical Oncology Unit, Department of Internal Medicine, Oncology Center, Faculty of Medicine, Mansoura University, Egypt. *

**Keywords:** Acute myeloid leukemia, SDF-1, CXCR4, PCR-RFLP

## Abstract

**Background:**

Acute myeloid leukemia (AML) is a heterogenous group of disorders that emerge from the malignant transformation of hematopoietic stem cells. Chemokine stromal cell-derived factor 1(SDF-1) and its receptor CXC receptor 4 (CXCR4) has an essential role in dissemination of blast cells. Study aimed to detect CXCR4 expression and the *SDF-1* (*rs1801157*) gene polymorphisms and correlate them with prognosis and outcome in AML patients.

**Subjects and Methods:**

The study was conducted on 60 de-novo AML patients, and 60 healthy controls. *SDF-1* (*rs1801157*) gene polymorphisms were detected by polymerase chain reaction-restriction fragment length polymorphism (PCR-RFLP), and CXCR4 expression was done using flow cytometry analysis.

**Results:**

SDF-1 dominant model (AG+AA) had higher risk AML (p 0.002). CXCR4 positive cases were associated significantly with toxic manifestations (p 0.019), lower CR rates (p 0.004), and unfavorable cytogenetics (p 0.027). Multivariate analysis showed that combined CXCR4positive with dominant SDF-1 considered as independent prognostic factor for shorter overall survival (OS) in AML patients (p 0.031).

**Conclusion:**

SDF-1 dominant model had a higher risk to develop AML, and CXCR4 positive expression predicts poor prognosis in AML patients and it could represent a targeted therapy in AML. In addition, CXCR4 could be easily integrated into the initial routine diagnostic work up of AML.

## Introduction

Acute myeloid leukemia (AML) is a heterogenous group of disorders, in which the blast cells either not, minimally, or abnormally differentiated (Tuzuner and Bennett, 2018). AML patients although initially responsive to chemotherapeutic agents, generally have a poor outcome and eventually will relapse from minimal residual disease (MRD). A better understanding of leukemia biology is needed to identify new prognostic markers and to choose targeted therapeutic options (Thakral and Gupta, 2019).

 One of new molecular targets for therapy and biological markers of prognosis is the CXC chemokine receptor 4 (CXCR4) which is one of chemokine receptors defined by their ability to induce cell migration towards a chemotactic cytokine gradient (chemotaxis) (Mollica et al., 2019; Du et al., 2019).

CXCR4 has an important role as it is the receptor for stromal derived factor (SDF-1), also known as CXC ligand 12 which is a member of chemokine family and a major molecule governing hematopoietic stem cells (HSCs) maintenance, regulating the process of embryogenesis, neutrophil homeostasis, inflammatory response, tumorigenesis and infiltration (Peled et al., 2018; Pillozzi et al., 2019).

Several recent studies have shown that SDF-1/CXCR4 pathway can play an important role in the progression of tumor (Fujita et al., 2018; Meng et al., 2018; Cho et al., 2017). Malignant cells express high levels of CXCR4, and metastasis target organs express high levels of SDF-1, allowing tumor cells to migrate to target organs via SDF-1/CXCR4 pathway chemotaxis. In AML patients, the surface of leukemia cells expresses high levels of CXCR4, which is a key molecule involved in anchoring leukemic HSCs within the bone marrow environment (Peled et al., 2018).

The present study aimed to study *SDF-1* gene polymorphisms at position 801(G to A, rs 1801157) and expression of *CXCR4* and to correlate them with the clinical characteristics, prognosis and outcome in AML patients.

## Materials and Methods

*Subjects*

A study was conducted on 60 patients with de novo acute myeloid leukemia patients were diagnosed at the Oncology Center Mansoura University (OCMU). Their ages ranged between 19 and 70 years and apparently healthy 60 individuals were also included as a control group. All data were collected regarding history and examination. Laboratory procedures were performed in clinical pathology labs of OCMU. Induction therapy was based on age and performance status as fit patients treated by 3+7 and consolidation by high dose Ara C, while frail one by low dose cytarabine or hypomethylating agents. The study excluded the patients with acute promyelocytic leukemia, secondary AML and childhood AML. 

A) Routine work: (1) Complete blood count (Hemogram): using the electronic counter (CELL-DYN 3700, Abbott, Canada) with thorough examination of peripheral blood smears stained with leishman stain. (2) Liver function tests, serum creatinine, serum uric acid test, and serum LDH.

B) Work up for AML diagnosis: (1) Microscopic study of bone marrow slides. (2) Cytochemistry: e.g. MPO, PAS, and NSE. (3) Immunophenotyping (IPT) using American BD flow-cytometer device to diagnose the cases and exclude the other types of leukemia by using cell surface marker including myeloid and lymphoid lineage. Positivity in each marker can be calculated if it is more than 20%. (4) Fluorescence in situ hybridization (FISH).

C) Specific work up: (1) Detection of (*SDF-1 G801A, rs 1801157*) gene polymorphisms by polymerase chain reaction-restriction fragment length polymorphism (PCR-RFLP) assay (for patients and controls). (2) Detection of *CXCR4* receptor expression by flow cytometry (for patients only).

*Methods*

I- Detection of (*SDF-1 G801A, rs 1801157*) gene polymorphisms by PCR-RFLP

Sampling: Two milliliters put in tube containing anticoagulant (EDTA) to genomic DNA extraction preparing for investigation (*SDF-1 G801A, rs 1801157*) polymorphisms by PCR-RFLP test.

DNA extraction: Done by Gene JET Whole Blood Genomic DNA Purification Mini Kit (#K0781).

PCR reaction: The test was done in 3 steps: (1) Amplification of extracted genomic DNA. (2) The amplified product is then digested by a suitable restriction enzyme. (3) Detection of the digested products using gel electrophoresis and ultraviolet light transillumination. 

The electronic warm cycler might have been programmed to the emulating states to *SDF-1 G801A *gene polymorphisms those accompanying cycles were utilized: a beginning high temperature –activation step at 95 °C for 10 minutes, denaturation toward 95°C to 1 min then last development in 72°C for 10 minutes.

- For (*SDF-1 G801A, rs 1801157*) gene polymorphisms, the following primers were used:

- Forward primer: 5′ CAG TCA ACC TGG GCA AAG CC 3′.

- Reverse primer: 5′ AGC TTT GGT CCT GAG AGT CC 3′.

- PCR products were digested at 37°C with 10U MspI resulting in generation of two fragments of 202-bp and 100-bp after digestion in the presence of the G allele and it is designated GG, wild type (i.e. homozygous for the presence of restriction site), if the A allele exists at position 801, no digestion occurred and only one 302 bp band will emerge and it is designated AA (i.e. homozygous for the absence of the restriction site), if the 3 bands 302,202and 100 bp are present it is designated AG (i.e. heterozygous).

II- Detection of *CXCR4* receptor expression by Flow cytometry and a cut off equals to 20.0% was used.

Sampling Bone marrow aspirate or venous blood samples examined for *CXCR4* expression by flow cytometry (BD FACSCANTOII), generally within 2 to 4 hours after the sample was drawn.

principle: Washed cells were incubated with the fluorescein labeled monoclonal antibody [Phycoerythrin (PE) Mouse Anti-Human CD184 monoclonal antibody (#561733) (BD Biosciences, San Diego, CA)], which attach to the cells with express the CXCR4 receptor. Unbound fluorescein-conjugated antibodies were at that point washed from the cells. Cells with express the CXCR4 receptor are fluorescently stained, with the intensity of staining directly proportional to the density of the CXCR4. Cell surface expression of the* CXCR4 *was decided by cytometric examination using 488 nm wavelength laser excitation.

*Statistical Analysis*

The statistical analysis of data was done using SPSS (statistical package for social science) program (SPSS, Inc, Chicago, IL) version 20. Qualitative data were presented as frequency and percentage. Chi square and Fisher’s exact tests were used to compare groups. Quantitative data were presented by mean, SD or median and range. Comparisons between two groups were done using t-test or Man Whitney (for non-parametric data), while comparison between more than two groups were done using ANOVA or Kruskal Wallis tests (for non-parametric data). The OR and 95% confidence interval (95% CI) obtained from the logistic regression were used Kaplan–Meier test was used for survival analysis and the statistical significance of C among curves was determined by Log-Rank test. Cox regression analysis was used for prediction of OS. P value is significant if <0.05 at confidence interval 95%.

*Sample size*

Sample size is calculated using online sample size calculator (http://osse.bii.a-star.edu.sg/calculation1.php) with genotype polymorphism of *SDF1* (Aisha et al., 2010) and level of absolute precision of 2% at alpha error of 5% and study power of 80%. A minimal sample size required for the study is calculated to be 54 subjects for each group. To account for possible, drop out a total sample of 60 subjects in each group is initially planned to be included in the study.

## Results

Our study included 60 new AML cases and 60 healthy control. Comparison between control and AML patients are summarized in Table 1. 

**Figure 1:**
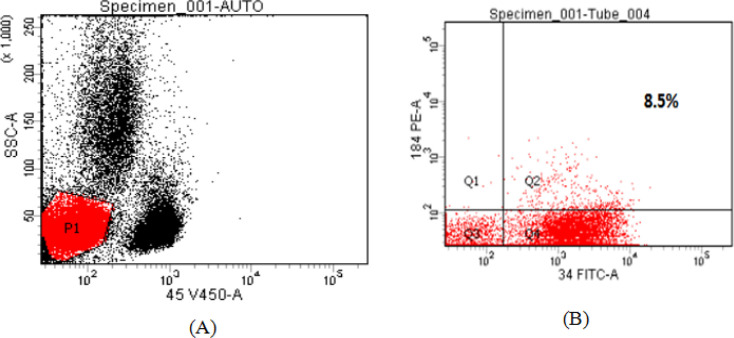
Negative Flow Cytometric Analysis of *CXCR4 *Expression on AML Myeloblast Cells. These results were obtained from a patient with newly diagnosed acute myelomonocytic leukemia (FAB-M4 AML). CXCR4(CD184 PE) expression was measured on myeloblast populations after setting a gate on side scatter (SSC)/anti-CD45 V450 scatter graph, completed with CD34 expression to optimize myeloblast gating. CXCR4 expression was negative in this case =8.5%.

**Figure 2:**
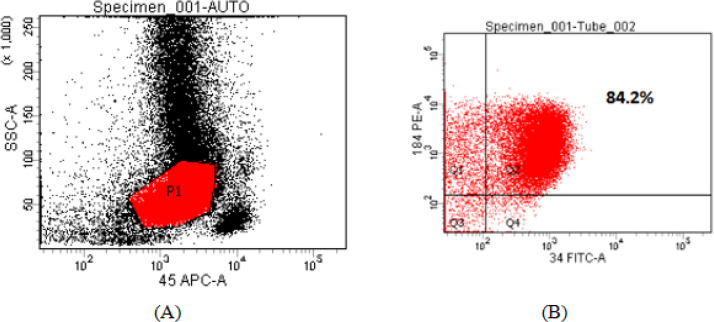
Positive Flow Cytometric Analysis of *CXCR4* Expression on AML Myeloblast Cells. Flow cytometric analysis of CXCR4 expression on AML myeloblast cells. These results were obtained from a patient with newly diagnosed acute myeloblastic leukemia (FAB-M2 AML). CXCR4(CD184 PE) expression was measured on myeloblast populations after setting a gate on side scatter (SSC)/anti-CD45 APC scatter graph, completed with CD34 expression to optimize myeloblast gating. expression was positive in this case =84.2%.

CXCR4 was measured by flow cytometry and a cut off equals to 20.0% was used, so that values ≥ 20.0% were considered CXCR4 positive, while those below this level were considered CXCR4 negative Table (2). Negative and Positive flow cytometric analysis of *CXCR4* expression on AML myeloblast cells are shown in Figures 1 and 2.

**Figure 3:**
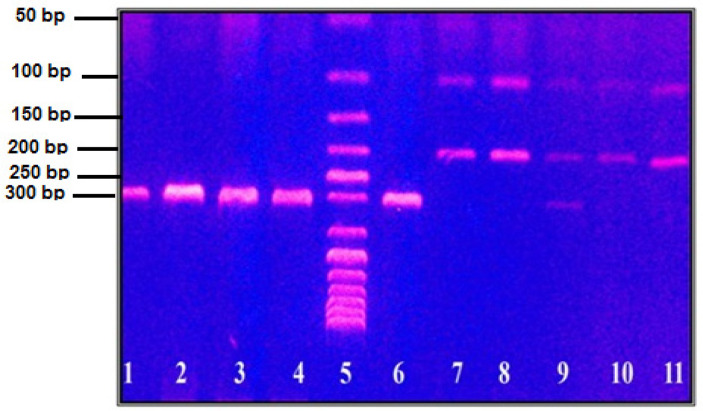
RFLP aAnalysis of *SDF-1 *Gene Polymorphism in AML Patients. Lane 5 is a 50 bp DNA marker. From lane 1 to 4 are a PCR product of SDF-1 gene at 302 bp. Lane 6 is homozygous AA genotype at 302 bp. Lane 7,8,10 and 11 are homozygous GG genotype at 100 and 202 bp. Lane 9 is heterozygous AG genotype at 100, 202 and 302 bp

SDF-1 (A) and (G) allele carrier frequencies show significant differences in patients versus controls (p 0.002). Carrying A allele had higher risk to develop AML (OR 3.134, 95% CI 1.521-6.459). When combining both hetero- and homozygous (AG+AA) groups; there was significant difference in patients versus control subjects (p 0.002). SDF-1(rs1801157) dominant model (AG+AA) had higher risk to develop AML (OR 3.500, 95%CI=1.555-7.874) Table (3). RFLP analysis of *SDF-1 *(*G801A, rs 1801157*) gene polymorphisms in AML patients is shown in Figure 3. 

CXCR4positive cases were associated significantly with toxic manifestations (p 0.019) and lower CR rates (p 0.004) compared to those with CXCR4negative. Notably, it was found that 75 % of CXCR4positive and 64.3% of SDF-1 (A) allele genotype were AML M4/M5 patients. Patients with positive CXCR4 had higher unfavorable cytogenetics when compared to those with negative CXCR4 (58.3% vs 27.8%, p 0.027). No statistically significant differences between different SDF-1 genotypes as regard clinical presentation, cytogenetic risk stratification, and outcome Table 4.

When stratifying AML into 4 groups according CXCR4 positivity and* SDF1* gene polymorphisms, CXCR4positive with SDF-1 (A) allele carrier genotype AML patients had significantly higher incidence of toxic manifestations (p 0.023) and lower CR (p 0.016) versus CXCR4negative with SDF-1 (G\G) alleles genotype AML patients and disconcordant respectively. Otherwise, no significant differences were found regarding other parameters. As regard clinical outcome of AML cases according to combined CXCR4 positivity and SDF-1 genotypes, no significant differences between the 4 groups and between CXCR4 negative with SDF1 (G\G) alleles genotype AML patients versus disconcordant versus CXCR4 positive with SDF-1(A) allele carrier genotype AML patients were found in clinical outcome Table 5.

**Figure 4:**
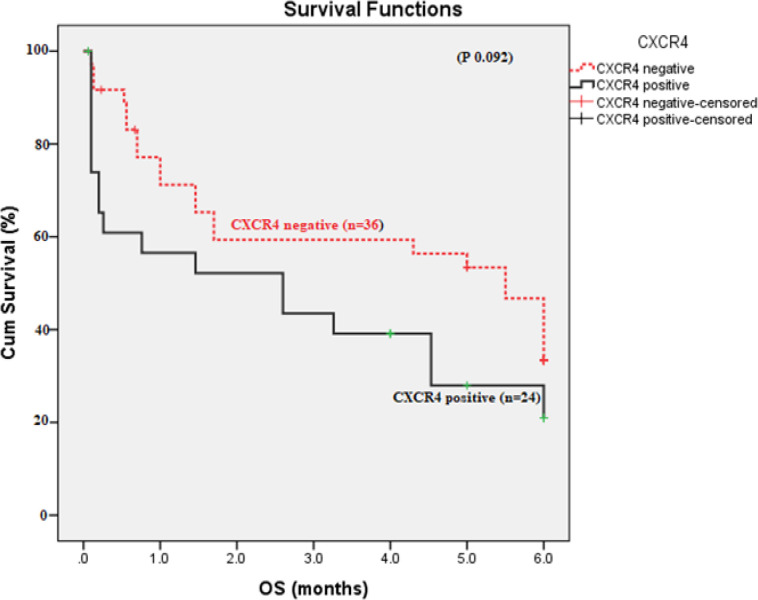
Overall Survival According to *CXCR4* Expression in AML Patients (p 0.092).

There was no statistically significant difference in OS and DFS as regarding *CXCR4* expression (p 0.092 and 0.9 respectively). In addition, there was no significant difference was found in OS between SDF1 GG versus SDF1 AG+AA genotypes (p 0.113). Patients with CXCR4positive and SDF1 (AA /AG) had higher risk to have shorter OS (HR 3.891, 95% CI 1.389-10.899, p 0.01) Figure (4and5). Patients with favorable cytogenetics had longer OS than those intermediate and unfavorable cytogenetics (5.418 versus 2.358, 2.111 months respectively, p 0.001), Figure 6.

Prediction of survival was done using cox regression analysis applying age, marrow blasts, and combined *CXCR4* expression and *SDF-1* genotypes as covariates and cytogenetics. Multivariate analysis showed that combined CXCR4 positive with SDF-1 (AG + AA) which reached statistical significance and considered as independent prognostic factor for AML patients (p 0.031). Patients with positive CXCR4 and SDF-1 (AG+AA) had higher risk to have shorter OS (HR 4.274, 95% CI 1.161-15.738). Age is still also independent risk factor for shorter OS (p 0.043). Also, intermediate and unfavorable outcome are independent risk factors for shorter OS (HR 3.701, 95% CI 1.544-8.871, p 0.003), (HR 4.165, 95% CI 1.184-9.427, p 0.001) respectively Table 6. 

**Table 1:** Comparison between Age, Gender and Hematological Data at Diagnosis of Different Studied Groups

Covariates	Median (Range)		P
Age (years)	45.5 (21-69)	43 (19 -70)	0.624
Gender	No. (%)		P
Males	33 (55%)	30 (50%)	0.715
Females	27 (45%)	30 (50%)	
Hematological data	Median (Range)		P
Total leucocytic count (x10^9^/l)	7.9 (5-10)	60.5 (1.0- 236)	*<0.001*
Hemoglobin concentration (g/dl)	13.1 (11.5-15)	7.7 (3.8-13.7)	*<0.001*
Platelet count (x10^9^/l)	262.5 (170-350)	33 (8.0-180)	*<0.001*
Absolute neutrophilic count (x10^9^/l)	6.5 (5-22)	4.7 (0.0006 -50)	*0.031*

**Table 2:** *CXCR4* Expression in AML Patients

Items		Number	%
CXCR4 expression	CXCR4	CXCR4^negative^	CXCR4^positive^
	Number of cases	36	24
	% of cases	60%	40%
	Range	2-18	21 – 84
	Median	7.5	44.5

**Table 3:** Genotypes and Alleles Frequencies of the SDF-1 Polymorphisms and Risk of AML

SDF1		Control (n=60)		AML (n=60)		P	OR	95% CI
		N.	%	N.	%			
Genotype	GG	48	80	32	53.3		1	reference
	AG, AA	12	20	28	46.7	0.002	3.5	1.555-7.874
Allele	G	108	90	89	74.2		1	reference
	A	12	10	31	25.8	0.002	3.134	1.521-6.459

CI 95%: Confidence interval at 95%.

**Table 4:** Clinical Characteristics of Studied AML Cases According to *CXCR4* Positivity and *SDF-1* Genotypes

Clinical data	CXCR4 expression				P	SDF-1 genotypes				
	Negative (n=36)		Positive (n=24)			GG (n=32)		(A) allele carrier (n=28)		p
	No	%	No	%		No	%	No	%	
Age										
Age <60	29	80.6	23	95.8	0.128	26	81.3	26	92.9	0.264
Age ≥60	7	19.4	1	4.2		6	18.8	2	7.1	
TLCx10^9^/l										
Median	54		64		0.251	57		59		0.87
Min - Max	1-236		1-214			3-207		1-236		
BM blast cells										
Median	92		93.5		0.221	90		92		0.3
Min - Max	23-100		28-100			23-100		33-99		
FAB classification										
M1/M2	12	33.3	6	25	0.145	10	31.3	8	28.6	0.97
M4-5	20	55.6	18	75		20	62.5	18	64.3	
M7	4	11.1	0	0		2	6.3	2	7.1	
Clinical presentation										
Toxic manifestations (n=34)	16	44.4	18	75	0.019	15	46.9	19	67.9	0.102
Extramedullary infiltration (EMI) (n=51)	28	77.8	23	95.8	0.072	27	84.4	24	85.7	0.885
Cytogenetics risk stratification										
Favorable	17	47.2	4	16.7		12	37.5	9	32.1	0.488
Intermediate	9	25	6	25		6	18.8	9	32.1	
Unfavorable	10	27.8	14	58.3	0.027	14	43.8	10	35.8	
Response rate										
CR	16	44.4	2	8.3	0.004	8	25	10	35.7	0.366
Non-CR (RD-ID)	20	55.6	22	91.7		24	75	18	64.3	

CR, Complete response; ID, Induction death; RD, Refractory disease; TLC, Total leucocytic count.

**Table 5:** Clinical Characteristics of Studied AML Cases According to Combined CXCR4 Positivity and *SDF-1* Gene Polymorphisms

Clinical data	CXCR4^negative^ and SDF-1 GG (n=17)		CXCR4^negative^ and SDF-1 AG+AA (n=19)		CXCR4^positive^ and SDF-1 GG (n=15)		CXCR4^positive^ and SDF-1 AG+AA (n=9)		P*	P^
	No	%	No	%	No	%	No	%		
Age										
Age <60	12	70.6	17	89.5	14	93.3	9	100	0.117	0.055
Age ≥60	5	29.4	2	10.5	1	6.7	0	0		
TLC x 10^9^/l										
Median	33		57		64		31		0.391	0.296
BM blast cells										
Median	90		92		92		95		0.382	0.219
FAB										
M1/M2	6	35.3	6	31.6	4	26.7	2	22.2	0.675	0.693
M4-5	9	52.9	11	57.9	11	73.3	7	77.8		
M7	2	11.8	2	10.5	0	0.0	0	0		
Clinical presentation										
Toxic manifestations (n=34)	5	29.4	11	57.9	10	66.7	8	88.9	0.023	0.01
EMI (n=51)	12	70.9	16	84.2	15	100.0	8	88.9	0.137	0.143
Cytogenetics risk stratification										
Favorable (n=21)	9	53	8	42.1	3	20.0	1	11.2	0.099	0.174
Intermediate (n=15)	4	23.5	5	26.3	2	13.3	4	44.4		
Unfavorable (n=24)	4	23.5	6	31.6	10	66.7	4	44.4		
Response rate										
CR	8	47.1	8	42.1	0	0.0	2	22.2	0.016	0.193
Non-CR (ID and RD)	9	52.9	11	57.9	15	100	7	77.8		

P*, significance between 4 groups; p^, significance between CXCR4 negative with SDF-1(G\G) alleles genotype AML patients vs disconcordant vs CXCR4 positive with SDF-1(A) allele carrier genotype AML patients; CR, Complete response; ID, Induction death; RD, Refractory disease; EMI, Extra medullary infiltration.

**Table 6:** Multivariate Analysis for OS and DFS in AML Patients

Covariates		OS			DFS		
		P	HR	95% CI	P	HR	95% CI
Age (years)		0.043	1.032	1.001-1.065	0.245	0.832	0.609-1.135
Marrow blast (%)		0.052	1.027	0.998-1.054	0.585	1.021	0.948-1.099
Combined *CXCR4* expression & SDF-1 polymorphism	CXCR4^negative ^and SDF-1 GG	-	1	Reference	-	1	Reference
	CXCR4^negative ^and SDF-1 AG+AA	0.071	2.712	0.917-8.026	0.132	2.877	0.202-2.119
	CXCR4positive and SDF-1 AA	0.111	2.587	0.804-8.326	0.18	1.703	0.055-5.226
	CXCR4positive and SDF-1 AG+AA	0.031	4.274	1.161-15.738	0.872	0.575	0.001-4.133
Cytogenetic risk stratification	Favorable	-	1	Reference	-	1	Reference
	Intermediate	0.003	3.701	1.544-8.871	0.135	0.956	0.784-1.125
	Unfavorable	0.001	4.165	1.840-9.427	0.834	0.849	0.183-3.937

CI 95%, Confidence interval at 95%; OS, Overall survival; DFS, Disease free survival; HR, High risk.

**Figure 5:**
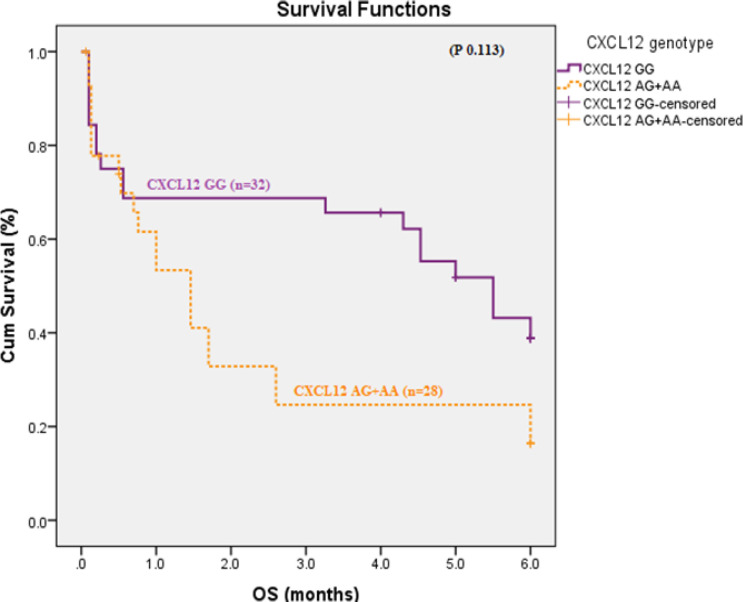
Overall Survival According to SDF-1 (GG vs AG+AA) Genotypes in AML Patients (p 0.113).

**Figure 6:**
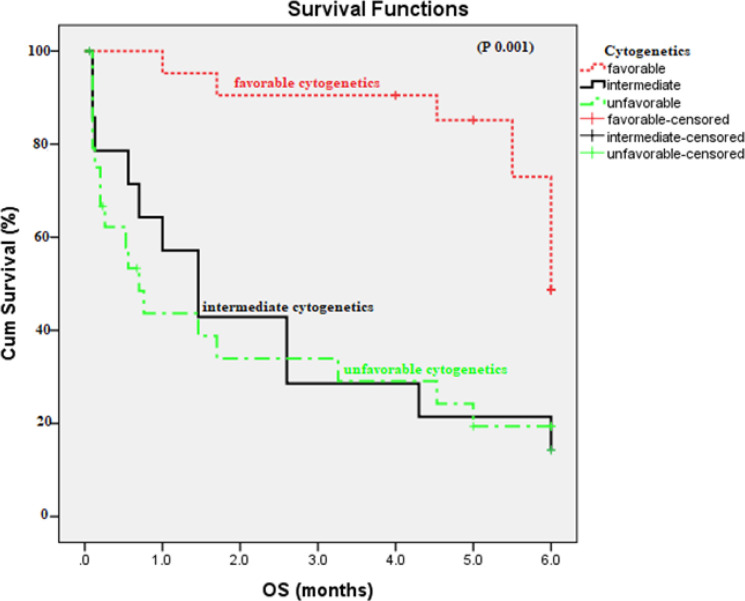
Overall Survival According to Cytogenetics Risk Stratification in AML Patients (p 0.001).

## Discussion

The interaction between leukemia cells and the bone marrow (BM) microenvironment is considered the major factor for development of chemotherapy resistance and disease relapse in leukemia. The CXCR4/SDF-1α axis played an essential role in the crosstalk between leukemia cells and BM microenvironment (Gualberto et al., 2017). 

Binding of SDF-1α to its cognate receptor CXCR4 on blast cell regulates blast cells trafficking. Blast cells are recruited and reside in the BM, thereby acquire anti-apoptosis signals and favorable conditions for growth and survival (Hu et al., 2018). 

*CXCR4* expression has been well studied in a different hematological and non- hematological malignancies e.g. chronic lymphocytic leukemia, myelodysplastic syndrome, multiple myeloma, pediatric AML, lung cancer, ovarian cancer, and breast cancer, in which it has been associated with adverse clinical outcomes, invasion, chemotherapy resistance, and metastasis (Cao et al., 2019). SDF-1 SNP researches have been conducted for several cancer types, but the results were conflicted based on the different studied population races, and cancer biology (Du et al., 2019).

The aim of this work is to study the expression of *CXCR4* by flow cytometry and the* SDF-1* gene polymorphisms by PCR-RFLP, and its relation to the clinical characteristics, patient prognosis and outcome. 

In our study, there was significant difference between *SDF-1 (rs 1801157)* genotype expression in AML group and control subjects, as SDF-1 dominant model (AG or AG+AA) had higher risk to develop AML (p 0.002). The results of the current study cope with other meta- analysis studies reported by Meng et al., (2015); Zhang et al., (2017) and Tong et al., (2016) that found also association between *SDF-1 G801A* polymorphisms and development of hematological malignancies especially AML. In contrast; Zheng et al., (2016); El-Ghany et al., (2014) studied the* SDF-1 G801A* polymorphism in 466 and 48 adult de novo AML patients respectively, reported no significant difference in genotype distributions and allele frequency between AML patients and healthy controls.

As regard CXCR4, our studied cases were classified into 36 cases (60.0%) had negative *CXCR4* expression, their values ranged from 2 to 18; and 24 cases (40.0%) had positive *CXCR4* expression, their values ranged from 21 to 84. Abd El-Rahman et al., (2010), reported that, 55% of the patients were CXCR4positive while 45% were *CXCR4* negative expression on their blasts. The results were stated as the percentage of blasts co-expressing CXCR4 and CD34 within the gated population of blast cells with cut off value at 20%.

Our data showed no statistically significant differences regarding age and hematological laboratory data among CXCR4 positive and CXCR4 negative AML patients. While, toxic manifestations had significantly higher incidence in CXCR4 positive cases (p 0.019) compared to CXCR negative and also with combined CXCR4positive – SDF-1 (AA/AG) genotype AML patients (p 0.023) versus CXCR4negative, or CXCR4positive with SDF-1 (GG) genotype AML patients. Also, we found that that 75% of positive CXCR4 and 64.3% of SDF-1 (A) allele genotype were AML M4/5 patients, however p values were insignificant. Cao et al., (2019) showed that patients with FAB subtypes M4/5 had significantly higher* CXCR4* expression than patients with other FAB subtypes.

Our results showed that CXCRpositive and Combined CXCRpositive and SDF1-(A) allele carrier genotype AML patients were associated with higher frequency of extra medullary infiltration (EMI) than the other group, however, p values were insignificant; 0.07, 0.137 respectively when compared to other groups. Similar Studies on acute leukemia showed that CXCR4 positivity and polymorphism in the* SDF-1* coding gene (*SDF-1 G801A)* were associated with a higher frequency of extramedullary leukemic infiltration (Crazzolara et al., 2001; Liu et al., 2006).

The present study showed that patients with CXCR4 positive had unfavorable cytogenetic risk and lower CR rates compared to those with CXCR4negative (p 0.027 and 0.004 respectively). Also, Abd El-Rahman et al., (2010) demonstrated that, in CXCR4positive group, 73% of patients had unfavorable prognosis compared to 27% in CXCR4negative group. 

 Du et al., (2019) reported that SDF1/ CXCR4 axis may protect the leukemia cells from the chemotherapy effect by intrinsic immune regulation, through secretion of cytokines that suppress apoptosis and decreasing the sensitivity to chemotherapy. 

Disruption of SDF-1/CXCR4 pathway is a key step in cytokine-induced hematopoietic stem cells mobilization from BM to peripheral blood. Plerixafor is a novel strategy of sensitizing myeloblast cells by targeting their protective BM microenvironment (Domingues et al., 2017). 

In our study, prediction of survival by multivariate analysis showed that combined positive CXCR4 and SDF-1 dominant model (AG+AA) had higher risk to have shorter OS (HR 4.274, 95% CI 1.161-15.73, p 0.031) and age is still independent risk factor for shorter OS. Also, intermediate and unfavorable cytogenetic risk are independent risk factors for shorter OS (HR 3.701, 95% CI 1.544-8.871, p 0.003), (HR 4.165, 95% CI 1.184-9.427, p 0.001) respectively.

In multivariate Cox regression analysis done by Spoo et al., (2007) only unfavorable cytogenetics and *CXCR4* expression by AML cells were significant predictive factors for poor OS. Also, *CXCR4* expression was a significant predictor for relapse free survival. Similarly, Konoplev et al., (2007), proved the prognostic value of *CXCR4* expression to be independent of other previously established prognostic markers such as age.

From this study, we could conclude that SDF-1 dominant model (AG+AA) had a higher risk to develop AML and that *CXCR4* expression predicts poor prognosis in AML patients and it could represent a novel target for the effective treatment of AML. Finally, CXCR4 could be easily incorporated into the initial routine diagnostic work-up of AML patients.

## Author Contribution Statement

Doaa Abd Allah M Aladle, Metwaly Ibrahim Mortada, Mayada A. Ghannam -equally participated in conception, interpretation of laboratory data and practical work. Shaimaa El-Ashwah, F.E.I. Ghobrial- collecting clinical data and statistical analysis. All authors - contributing to the study design, participated in writing and editing the final version of the manuscript, read and approved the final manuscript .

## Ethics approval

This study was approved by the institutional research committee of the investigating hospital Institutional Review Board (IRB), Mansoura Faculty of Medicine, number (R/19.02.436) and the 1964 Helsinki Declaration and its later amendments or comparable ethics standards. 

## Conflict of interest

The authors declare that there is no any conflict of interest.
